# A simplified workflow for monoclonal antibody sequencing

**DOI:** 10.1371/journal.pone.0218717

**Published:** 2019-06-24

**Authors:** Lena Meyer, Tomás López, Rafaela Espinosa, Carlos F. Arias, Christopher Vollmers, Rebecca M. DuBois

**Affiliations:** 1 Department of Biomolecular Engineering, University of California Santa Cruz, Santa Cruz, California, United States of America; 2 Departamento de Genética del Desarrollo y Fisiología Molecular, Instituto de Biotecnología, Universidad Nacional Autónoma de México, Cuernavaca, Morelos, México; University of Lincoln, UNITED KINGDOM

## Abstract

The diversity of antibody variable regions makes cDNA sequencing challenging, and conventional monoclonal antibody cDNA amplification requires the use of degenerate primers. Here, we describe a simplified workflow for amplification of IgG antibody variable regions from hybridoma RNA by a specialized RT-PCR followed by Sanger sequencing. We perform three separate reactions for each hybridoma: one each for kappa, lambda, and heavy chain transcripts. We prime reverse transcription with a primer specific to the respective constant region and use a template-switch oligonucleotide, which creates a custom sequence at the 5’ end of the antibody cDNA. This template-switching circumvents the issue of low sequence homology and the need for degenerate primers. Instead, subsequent PCR amplification of the antibody cDNA molecules requires only two primers: one primer specific for the template-switch oligonucleotide sequence and a nested primer to the respective constant region. We successfully sequenced the variable regions of five mouse monoclonal IgG antibodies using this method, which enabled us to design chimeric mouse/human antibody expression plasmids for recombinant antibody production in mammalian cell culture expression systems. All five recombinant antibodies bind their respective antigens with high affinity, confirming that the amino acid sequences determined by our method are correct and demonstrating the high success rate of our method. Furthermore, we also designed RT-PCR primers and amplified the variable regions from RNA of cells transfected with chimeric mouse/human antibody expression plasmids, showing that our approach is also applicable to IgG antibodies of human origin. Our monoclonal antibody sequencing method is highly accurate, user-friendly, and very cost-effective.

## Introduction

Recombinant monoclonal antibodies (mAbs) are a multibillion-dollar industry.[1] In contrast to monoclonal antibodies generated using traditional hybridoma-based methods and isolated from ascites fluid, recombinant monoclonal antibodies are produced by cloning antibody cDNA or synthetic sequences into expression plasmids and expressing in mammalian cell culture.[2] Before the design of recombinant antibody expression plasmids, sequencing of the antibody light and heavy chain variable regions is required. These variable regions determine antigen binding. It is therefore critical to obtain the correct sequence of the variable regions to maintain antibody affinity and specificity. In addition, knowledge of the variable region sequences and subsequent recombinant antibody expression reduces the impact of hybridoma cell loss and hybridoma instability caused by mutations, chromosome deletions, or environmental factors.[3]

There are several existing methods to sequence antibody variable regions from hybridoma cells or lymphocytes. Some involve the use of high-throughput RNA-sequencing technologies.[4–6] These methods prove highly accurate and allow for the analysis of antibody repertoires to great depths.[6] However, most labs are not familiar with high-throughput sequencing technologies, which require expertise for the preparation of RNA-seq libraries and for computational analysis. Furthermore, the cost of high-throughput library preparation and sequencing can be substantial, and turn-around time at sequencing cores can be weeks to months.

Other methods to sequence antibody variable regions use PCR and Sanger sequencing.[7–13] Variable region sequence determination by PCR-based approaches is challenging due to difficulties in designing universal primers that amplify all possible variable region sequences. This problem arises as a result of the inherent low sequence identity in the variable regions themselves as well as in the 5’ leader sequence of antibody light and heavy chains, directly upstream of the variable regions.[14] Some approaches use sets of degenerate primers targeting the 5’ region to overcome this issue.[7–10] However, these degenerate primers sometimes result in amplification success rates of only 80–90% because of non-specific priming or no priming,[7, 10] meaning that 10–20% of antibody variable regions cannot be sequenced with these methods. An additional risk with degenerate primers is that the variable regions of the parent myeloma cell line can also amplify using these primers.[10] Other approaches use 5’ RACE (rapid amplification of 5’ cDNA ends),[11, 12] but mRNA degradation, cDNA purification, and polyA tail addition in between reverse transcription and PCR makes this approach somewhat tedious.[13] A technique using non-degenerate primers also exists, but each variable region needs multiple amplification attempts with different sets of primers as well as further sequence validation with mass spectrometry.[15] Furthermore, there is a non-negligible risk of introducing primer-derived mutations in these methods.

In addition to nucleic acid-based approaches, there are *de novo* protein sequencing approaches to determine antibody variable regions by mass spectrometry,[16–18] but these methods do not always lead to a single variable region sequence due to isobaric residues such as isoleucine and leucine.[19] A combination of X-ray crystallography and mass spectrometry positively identified variable region sequences.[20] However, this method is time-consuming, requires large amounts of purified monoclonal antibody, and is expensive.

Finally, researchers without access to these technologies may employ antibody sequencing services, such as those provided by GenScript, Syd Labs, Fusion Antibodies, or LakePharma.[21–24] Unfortunately, these services can become prohibitively expensive, costing at least $800 to sequence a single antibody’s variable regions. Here, we successfully implemented a robust, simple, and affordable approach to sequence monoclonal antibody variable regions from RNA with a turn-around time of five days at a cost of $70 –$120 per antibody.

## Results

### Monoclonal antibody sequencing strategy

To sequence the variable regions of five mouse monoclonal IgG1 antibodies (2D9, 3B4, 3E8, 3H4, and 4B6),[25] we extracted total RNA from the hybridoma cell lines expressing these antibodies and applied a modified RT-PCR (reverse transcription polymerase chain reaction) using SMART (switching mechanism at 5' end of RNA transcript) technology.[26, 27] This technology is based on the intrinsic features of the reverse transcriptase from the Moloney murine leukemia virus (MMLV) and the application of a custom-sequence template-switch oligonucleotide (template-switch oligo) forward primer containing 3 riboguanines (rGrGrG) at its 3' end. To amplify antibody variable regions, we designed the RT-PCR reverse primers to be specific for highly conserved sequences in the constant regions of kappa, lambda, and IgG heavy chains of mouse antibodies (Tables 1 and 2).

**Table 1:** Mouse IgG reverse transcription primers.

Primer Name	Forward or Reverse	Primer Sequence
Template-switch oligo	Universal forward primer	5’ aagcagtggtatcaacgcagagtacatgrgrgr 3’
mIGK RT	Reverse primer for kappa chain	5’ ttgtcgttcactgccatcaatc 3’
mIGL RT	Reverse primer for lambda chain	5’ ggggtaccatctaccttccag 3’
mIGHG RT	Reverse primer for heavy chain	5’ agctgggaaggtgtgcacac 3’

**Table 2:** Mouse IgG PCR primers.

Primer Name	Forward or Reverse	Primer Sequence
ISPCR	Universal forward primer	5’ aagcagtggtatcaacgcagag 3’
mIGK PCR	Reverse primer for kappa chain	5’ acattgatgtctttggggtagaag 3’
mIGL PCR	Reverse primer for lambda chain	5’ atcgtacacaccagtgtggc 3’
mIGHG PCR	Reverse primer for heavy chain	5’ gggatccagagttccaggtc 3’

**Fig 1:**
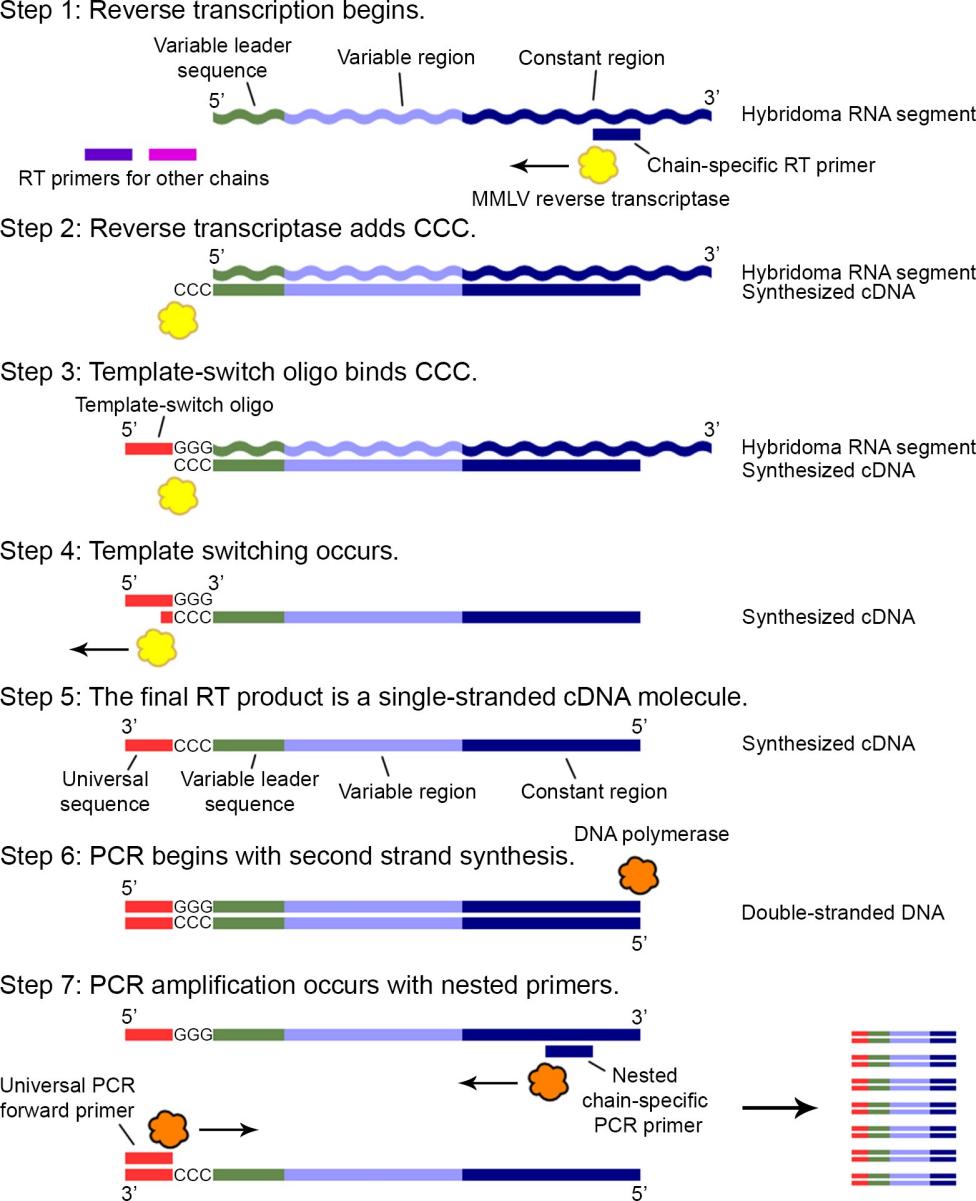
Schematic for cDNA synthesis by template-switching. (Step 1) Primer binding and initiation of polymerization. (Step 2) MMLV reverse transcriptase adds deoxycytosines to the cDNA 3' end. (Step 3) Template-switch oligo binds the CCC overhang. (Step 4) Reverse transcriptase switches templates and continues polymerization using the template-switch oligo as the template. (Steps 5–7) The single-stranded cDNA product of reverse transcription becomes the template for second-strand synthesis primed by the universal PCR forward primer. Amplification follows using the universal PCR forward primer and nested chain-specific PCR reverse primers. Note that the lengths of the different antibody regions and primers are not drawn to scale.

The RT-PCR amplification of antibody variable regions occurs as follows: To begin reverse transcription of a particular variable region, the reverse transcription primer specific for that antibody chain (either kappa, lambda, or heavy; Table 1) binds the hybridoma RNA within the constant region sequence at a highly conserved site. The MMLV reverse transcriptase initiates polymerization (Fig 1, Step 1). After the MMLV reverse transcriptase reaches the 5’ end of the RNA template during first strand synthesis, it adds several nucleotides, usually deoxycytosine, to the 3' end of the cDNA transcript (Fig 1, Step 2). The reliable addition of these bases by the MMLV reverse transcriptase allows annealing of the template-switch oligo (Table 1). When base pairing occurs between the template-switch oligo’s 3’ riboguanines and the cDNA deoxycytosine overhang (Fig 1, Step 3), the MMLV reverse transcriptase switches templates and continues polymerization, now using the template-switch oligo as the template rather than the hybridoma RNA, until it reaches the 5’ end of the template-switch oligo (Fig 1, Step 4). The final product is a single-stranded cDNA molecule containing an initial universal sequence added by the template-switch oligo followed by the complete 5’ to 3’ sequence of the RNA template region (Fig 1, Step 5). This cDNA becomes the template for second-strand synthesis (Fig 1, Step 6) and amplification in PCR (Fig 1, Step 7) by taking advantage of the added universal sequence. The forward PCR primer (Table 2) has the same sequence as the template-switch oligo and therefore binds the universal sequence added to the cDNA transcript during reverse transcription. The reverse PCR primers (Table 2) are again specific for each chain’s constant region at a second highly conserved sequence but are nested within the cDNA sequence synthesized during reverse transcription to promote amplification specificity.

**Fig 2:**
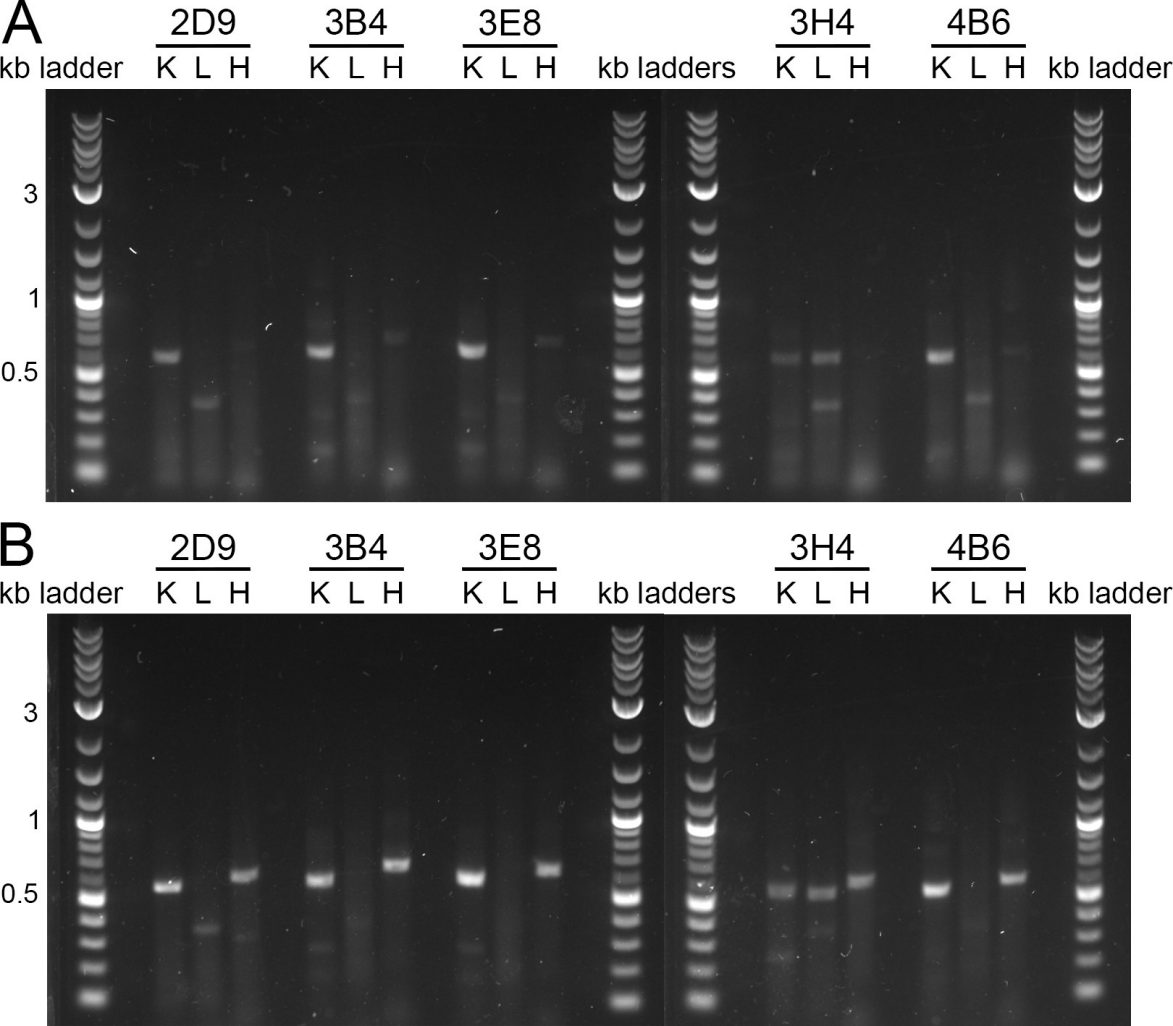
Comparison of primer sets for RT-PCR amplification of variable regions from 5 hybridoma mRNA samples. K = kappa chain, L = lambda chain, H = heavy chain. (A) RT-PCR result using the same reverse primers for RT and for PCR. (B) RT-PCR result using a set of nested reverse primers for RT and for PCR.

We set up fifteen total RT-PCR reactions: five hybridoma RNA samples with three RT-PCR reactions each to amplify kappa, lambda, and heavy chain variable regions. We set up kappa and lambda chain amplifications for each antibody because we did not know which light chain was present. We checked these reactions by agarose gel electrophoresis. After optimizing primer design (see the Primer selection section), 2D9, 3B4, 3E8, and 4B6 showed amplification of kappa chains, 3H4 showed amplification of both kappa and lambda chains, and all five samples showed amplification of heavy chains. Each amplicon is 550–600 base pairs in size (Fig 2B).

### Primer selection

To optimize RT-PCR amplification of the variable regions of mouse kappa, lambda, and IgG heavy chains, we designed and tested multiple sets of primers. We examined two strategies. For both strategies, we used the following primers for the RT step: the template-switch oligo forward primer and the reverse transcription primers specific for kappa, lambda, or heavy chain constant regions, called mIGK RT, mIGL RT, and mIGHG RT, respectively (Table 1). Following reverse transcription, we implemented different strategies for PCR amplification. For the first strategy, we used the same reverse primers for both RT and PCR; therefore, in the PCR step, we used the ISPCR forward primer (Table 2, Row 1), which has the same 5’ sequence as the template-switch oligo, and again used the mIGK RT, mIGL RT, or mIGHG RT reverse primers (Table 1). For the second primer strategy, the reverse PCR primers were nested within the sequence created by reverse transcription to promote specificity for the desired variable region amplicon (Fig 1, Step 7). In the PCR step in this strategy, we used the ISPCR forward primer and the nested reverse primers mIGK PCR, mIGL PCR, or mIGHG PCR (Table 2).

A comparison of the RT-PCR results obtained using the same reverse primers for RT and for PCR (Fig 2A) to the results obtained using nested primers (Fig 2B) clearly shows that nested primers produce better amplification of antibody variable regions. In particular, the heavy chains of each antibody did not amplify, or only amplified faintly, using the same reverse primers in RT and PCR but amplified well when choosing nested primers for PCR. In addition, using nested primers increased the intensity of the kappa amplification product for each antibody. Finally, use of the nested primers led to a reduction in intensity or even elimination of a ~350 base pair non-specific lambda chain amplicon. Therefore, we suggest using nested primers for monoclonal antibody sequencing.

We performed a Clustal Omega[28] multiple sequence alignment (not shown) of the constant regions from all subclasses of mouse IgG (IgG1, IgG2a, IgG2b, IgG2c, and IgG3) using sequences available on IMGT, the international ImMunoGeneTics information system.[29] Based on the alignment, we expect that mIGHG RT and mIGHG PCR can prime from the constant regions of IgG1, IgG2a, IgG2b, and IgG2c antibodies but likely not prime from the constant region of IgG3 antibodies because of five mismatches of the IgG3 constant region to each primer. Therefore, we predict that the primers given in Tables 1 and 2 may be used to sequence antibodies from the majority of all mouse IgG subclasses.

### Sequencing results

Following RT-PCR, we purified the amplicons by agarose gel extraction and directly sequenced by Sanger sequencing to determine the variable region sequences of the light and heavy chains from all five antibodies. We analyzed the sequencing data with a custom Python program available on GitHub. We identified the sequences of one kappa and three heavy chains in this manner (Table 3 under Number of Amplicons Sequenced). However, the sequencing data was not clear enough to determine the remaining sequences. Therefore, we turned to a sequencing vector to improve the quality of the DNA sequenced. We purified RT-PCR products with a PCR clean-up kit, blunt-end cloned into a plasmid, transformed into *E*. *coli*, and sequenced plasmid clones by Sanger sequencing. We obtained clear sequencing data for plasmid clones from each of the variable regions (Table 3). This result is an improvement on the outcome of direct PCR sequencing, which only positively identified four variable regions. We sequenced enough plasmid clones of each light and heavy chain variable region to compare at least three amino acid sequences of each variable region to confirm sequence identity (Table 3 under Number of Plasmid Clones Sequenced). We used Clustal Omega for these alignments (not shown).

**Table 3:** Results of sequencing RT-PCR products directly and following blunt-end cloning.

Antibody	Number of Amplicons Sequenced			Number of Plasmid Clones Sequenced		
	Total	Amplicons Containing Light Chain	Amplicons Containing Heavy Chain	Total	Clones Containing Light Chain	Clones Containing Heavy Chain
2D9	6	0	3	20	3	4
3B4	6	0	0	10	4	3
3E8	6	3	0	20	5	3
3H4	6	0	3	15	5	4
4B6	6	0	3	18	3	3

Once we identified a consensus sequence for each of the ten variable regions, we used the IgBLAST tool,[30] a tool for alignment of immunoglobulin and T cell receptor variable domain sequences, to determine percent identity of our light and heavy chain variable regions to IgBLAST reference sequences, i.e. the top-matched germline V genes. Table 4 shows the results of this query. Across the light and heavy chains we sequenced, percent identity to the reference ranges from 92.1% to 100% in the frame regions and ranges from 87.5% to 100% in the complementarity-determining regions (CDRs). All regions of 3E8 kappa, 4B6 kappa, 2D9 heavy, and 3E8 heavy chains match 100% to the reference sequences. For light chains, average percent identity of all frame regions and CDRs to the references is 99.2%. For heavy chains, average percent identity of all frame regions and CDRs to the references is 98%. We repeated this analysis using the IMGT database, which calculated the average percent identity of an antibody’s frame regions and CDRs combined instead of the percent identity of the individual regions. We also report these values in Table 4. These results support the conclusion that the sequences determined with our method are viable antibodies from hybridoma RNA.

**Table 4:** Percent identity to IgBLAST and IMGT reference sequences.

Antibody	Percent Identity of Light Chain							
	to IgBLAST References							to IMGT References
	Frame Region 1	Frame Region 2	Frame Region 3	CDR 1	CDR 2	CDR 3	Total	Total
2D9	100%	100%	100%	94.4%	100%	100%	99.7%	96%
3B4	100%	98%	99.1%	100%	88.9%	90%	98.2%	94%
3E8	100%	100%	100%	100%	100%	100%	100%	94%
3H4	100%	96.1%	98.1%	100%	100%	95.5%	98.3%	98%
4B6	100%	100%	100%	100%	100%	100%	100%	97%
Average	100%	98.8%	99.4%	98.9%	97.8%	97.1%	99.2%	95.8%
Antibody	Percent Identity of Heavy Chain							
	to IgBLAST References							to IMGT References
	Frame Region 1	Frame Region 2	Frame Region 3	CDR 1	CDR 2	CDR 3	Total	Total
2D9	100%	100%	100%	100%	100%	100%	100%	100%
3B4	97.3%	100%	94.7%	100%	91.7%	—	96.5%	96%
3E8	100%	100%	100%	100%	100%	100%	100%	99%
3H4	98.7%	96.1%	92.1%	91.7%	90.5%	87.5%	94.2%	93%
4B6	100%	100%	99.1%	100%	95.2%	100%	99.3%	99%
Average	99.2%	99.2%	97.2%	98.3%	95.5%	96.9%	98%	97.4%

Percent identity of each region is reported as given by IgBLAST or IMGT. The total percent identity was calculated by IgBLAST and IMGT as the number of matches between the query and reference sequences over the length of the aligned sequence multiplied by 100. The averages refer to the average (mean) of all values in that column.

**Fig 3:**
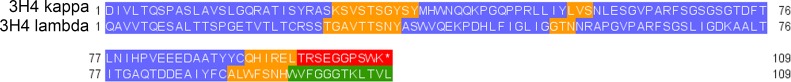
Protein sequence comparison of variable regions for 3H4 kappa and 3H4 lambda. Blue = Frame region, Orange = Complementarity-determining region, Red = J region out-of-frame, Green = J region in-frame 3H4 kappa (top) has an early stop codon due to a frameshift mutation. 3H4 lambda (bottom) is full-length.

Because amplification of both the kappa and lambda chains occurred for 3H4, as opposed to amplification of only one of the light chains as for the other four antibodies (Fig 2B), we purified the amplicons, blunt-end cloned, and sequenced the RT-PCR products for both 3H4 kappa and 3H4 lambda. Sequencing revealed that the 3H4 kappa variable region contains a frameshift mutation in the V-gene/J-gene junction, resulting in an early stop codon. In contrast, the 3H4 lambda variable region has a properly in-frame V-gene/J-gene junction and aligns to the reference sequence. Therefore, 3H4 lambda is likely the correct 3H4 light chain while 3H4 kappa represents an abortive rearrangement and might originate from a hybridoma fusion partner transcript.[31, 32] An amino acid sequence comparison of 3H4 kappa and 3H4 lambda can be seen in Fig 3, in which the alignment and coloring were performed with Jalview.[33] The values given in Tables 3 and 4 are for 3H4 lambda.

### Verification of antigen binding: Comparison of chimeric mAb 2D9 and mouse mAb 2D9

To validate our antibody sequences, we cloned, expressed, and purified five recombinant antibodies. Using the heavy chain and light chain amino acid sequences of each antibody, we engineered recombinant antibody constructs comprised of the variable regions from the original mouse antibody and the constant regions from the human IgG1 antibody VRC01.[34, 35] We refer to these constructs as chimeric monoclonal antibodies in this manuscript.

**Fig 4:**
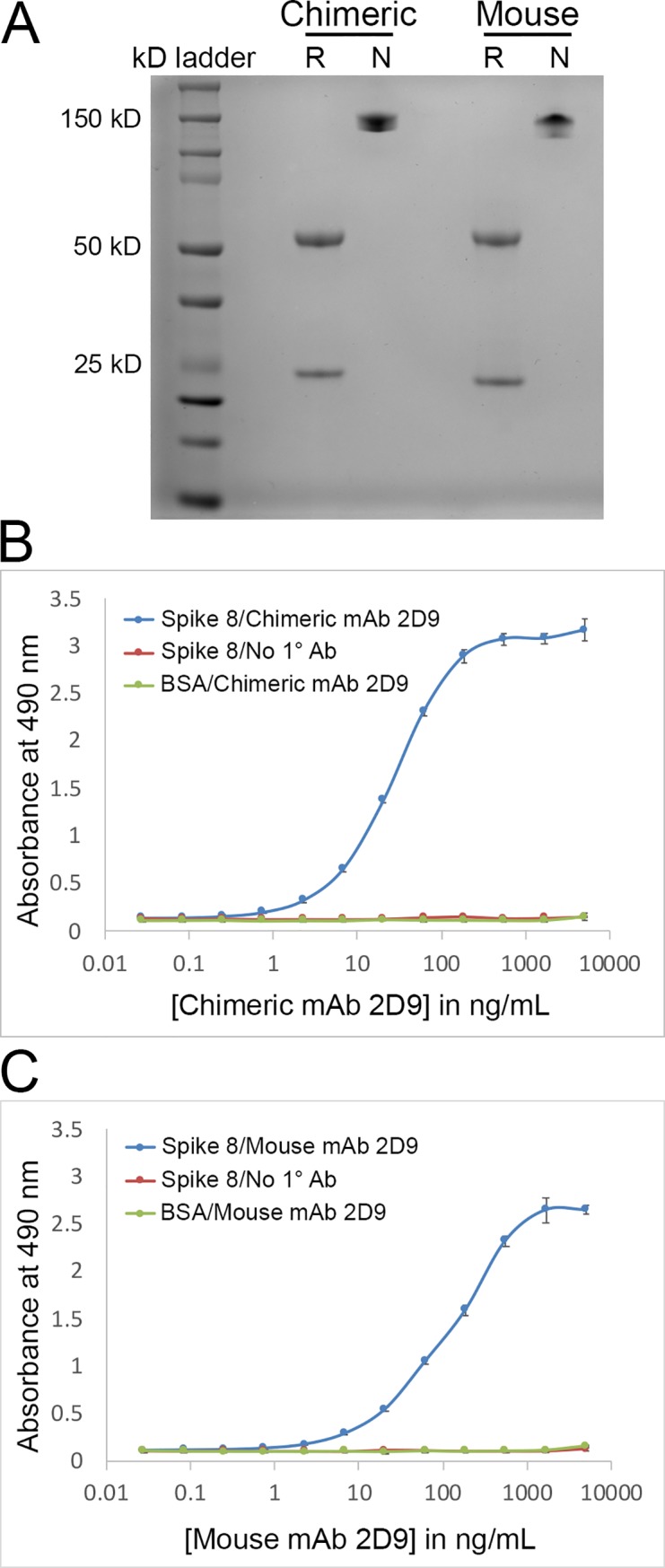
Comparison of chimeric mAb 2D9 and mouse mAb 2D9. R = reducing gel sample, N = non-reducing gel sample (A) SDS-PAGE gel comparing chimeric mAb 2D9 (left) to mouse mAb 2D9 (right). A reducing (R) and a non-reducing (N) sample is shown for each mAb. (B) Indirect ELISA showing that chimeric mAb 2D9 binds the Spike 8 antigen. (C) Indirect ELISA showing that mouse mAb 2D9 binds the Spike 8 antigen.

Using antibody 2D9 as a representative sample, we compared chimeric mAb 2D9 to its corresponding mouse antibody. Chimeric mAb 2D9 was transiently expressed in human embryonic kidney (HEK) 293F cells and purified from the medium by Protein A beads. Mouse mAb 2D9, the original antibody, was isolated from mouse ascites fluid and purified by Protein G beads. An SDS-PAGE gel comparison (Fig 4A) shows that both the original mouse mAb 2D9 and chimeric mAb 2D9 express light and heavy chains (reducing lanes) that form an antibody complex of the correct size, ~145 kD (non-reducing lanes).

Next, we tested the ability of the recombinant chimeric mAb 2D9 to bind its antigen, Spike 8, the recombinant capsid spike domain from human astrovirus serotype 8, against which mouse mAb 2D9 was raised.[25] By indirect ELISA, we found that both chimeric mAb 2D9 and mouse mAb 2D9 bind Spike 8 (Fig 4B and 4C). The other four chimeric mAbs also bind the human astrovirus spike against which they were raised. Thus, our simplified method of sequencing mouse IgG variable regions resulted in the correct sequences each time since all five recombinant chimeric antibodies constructed with those sequences retained their ability to bind the antigen used to raise the original mouse antibodies. This result indicates a performance rate of 100% for our method.

### Proof-of-concept: RT-PCR amplification of RNA from chimeric antibodies expressed in a human cell line

**Fig 5:**
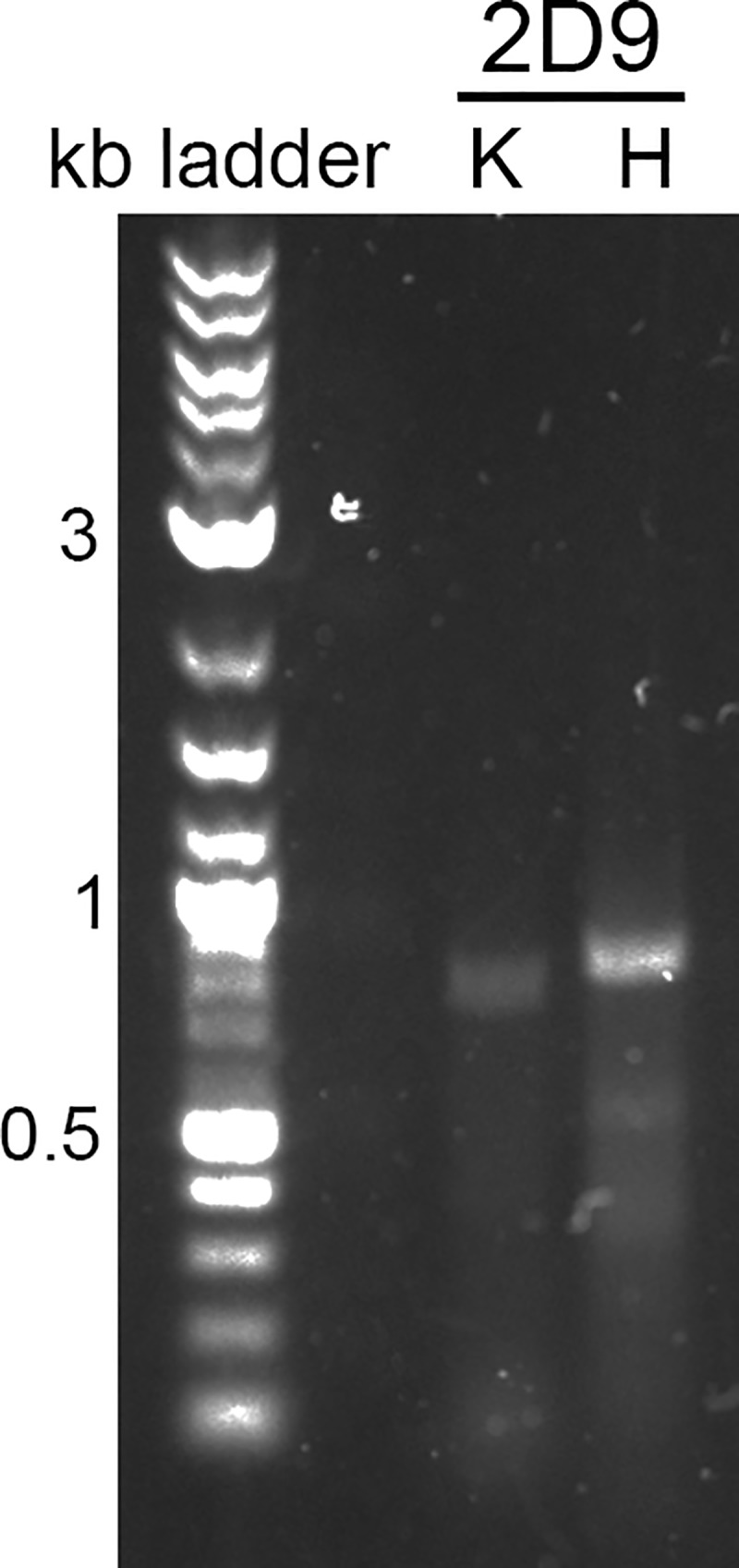
RT-PCR amplification of chimeric antibody variable regions. K = kappa chain, H = heavy chain RT-PCR result with reverse primers designed for human constant regions and using as a template the RNA extracted from HEK 293F cells transiently transfected with chimeric mAb 2D9 constructs.

Our success in amplifying and sequencing mouse antibody variable regions from hybridoma RNA led us to perform a proof-of-concept experiment and apply the same RT-PCR method, including cycle conditions, to RNA extracted from HEK 293F cells transiently transfected with chimeric mAb 2D9 plasmid constructs. We designed new RT-PCR reverse primers (Tables 5 and 6), which were this time specific for the constant regions of human IgG antibodies rather than mouse IgG antibodies. Since we now knew that mAb 2D9 contains a kappa chain and a heavy chain and does not have a lambda chain, we designed RT-PCR primers only for human kappa and IgG heavy chains. As shown in Fig 5, RT-PCR using the human primers on RNA extracted from HEK 293F cells was equally as successful as RT-PCR using the mouse primers on hybridoma RNA (Fig 2B). Therefore, we conclude that our method can be applied for the sequencing of human IgG variable regions as well as mouse IgG variable regions.

**Table 5:** Human IgG reverse transcription primers.

Primer Name	Forward or Reverse	Primer Sequence
Template-switch oligo	Universal forward primer	As above in Table 1
hIGK RT	Reverse primer for kappa chain	5’ gattggagggcgttatccacc 3’
hIGHG RT	Reverse primer for heavy chain	5’ gccgggaaggtgtgcacg 3’

**Table 6:** Human IgG PCR primers.

Primer Name	Forward or Reverse	Primer Sequence
ISPCR	Universal forward primer	As above in Table 2
hIGK PCR	Reverse primer for kappa chain	5’ tttggcctctctgggatagaag 3’
hIGHG PCR	Reverse primer for heavy chain	5’ agggcgcctgagttccacg 3’

As for mouse IgG subclasses, we performed a Clustal Omega[28] multiple sequence alignment (not shown) of the constant regions from all subclasses of human IgG (IgG1, IgG2, IgG3, and IgG4) using sequences available on IMGT.[29] We predict that hIGHG RT and hIGHG PCR can prime from the constant regions of human antibodies of all IgG subclasses because of a high degree of sequence conservation in the priming regions, with a maximum of one mismatch between the primers and a particular IgG subclass’ constant regions. Because of this high degree of conservation, we anticipate that our primers may be used to sequence antibodies from all human IgG subclasses.

We further expect that our method may be implemented to determine the variable region sequences of any antibody, provided that the proper constant region reverse primers for the antibody of interest are constructed. This feature makes our approach relevant for many users.

## Discussion

We have developed a straightforward method to sequence any variable region from any antibody, as long as constant region reverse primers for the proper chain are designed. We showed that this method works for mouse IgG1 and human IgG1 antibodies and have provided these primer sequences, which are expected to cover the majority of mouse and human IgG subclasses. We demonstrated that implementation of this method is uncomplicated; it requires only basic laboratory skills and equipment, which also contribute to the method’s affordability and rapidity. Compared to at least $800 to use an antibody sequencing service,[21–24] we estimate that it cost us $70 –$120 to sequence one antibody. Sanger sequencing, at approximately $5 a reaction, makes up about 80% of the cost and causes cost variation based on the number of plasmid clones sequenced. The duration of the method is as low as five days from RNA extraction to variable region sequence determination.

This method is broadly applicable. It can help researchers determine antibody sequences from brand-new hybridoma cell lines and from lymphocytes isolated from patients, thus allowing for production of recombinant antibodies and alternative antibody formats such as antibody-enzyme fusion proteins or bispecific antibodies. This method can also verify that old hybridoma cell lines still produce the proper antibody. Finally, application of this method ahead of time prevents loss of the antibody sequence if hybridoma cells cease to produce antibody when removed from frozen storage.

The RT-PCR basis of the method allows for sequence determination of multiple antibody samples at once by simply setting up several RT-PCR reactions in parallel. In addition, a small RNA sample is sufficient; each RT-PCR reaction was set up with only 100 ng of cellular RNA. Note that 45% of plasmid clones sent for sequencing contained DNA encoding antibody variable regions (Table 3). This percentage might be improved by purification of the RT-PCR product by gel extraction before blunt-end cloning rather than purification only by a PCR clean-up kit. Alternatively, Gibson assembly may be used to clone the RT-PCR products into a plasmid containing the template-switch oligo and reverse primer sequences.

Another advantage of this method is that it is not necessary to know if a monoclonal antibody contains a kappa or a lambda chain before RT-PCR amplification. Separate RT-PCR reactions can simply be set up for both types of light chain. In our case, for four of five antibodies, the correct light chain was clearly a kappa chain, as the lambda chain did not amplify. In the fifth case, Sanger sequencing distinguished between 3H4 kappa and 3H4 lambda and showed that 3H4 lambda chain is full-length. Thus, this method enables distinction between the two types of light chain.

## Conclusions

In summary, we successfully developed a simplified and affordable workflow to consistently determine antibody variable region sequences from RNA. First, we applied our method to RNA from five hybridomas producing mouse IgG1 monoclonal antibodies. All five recombinant chimeric antibodies engineered with the variable region sequences determined by our method preserved their ability to bind antigen, showing that our method is highly efficient and reliable in retrieving the sequences for a cognate light chain/heavy chain pair. We also showed that this method can be applied to IgG1 antibodies of human origin. Based on sequence conservation, we expect that the given primer sequences can amplify variable regions from the majority of all mouse IgG and human IgG subclasses. Other users may implement this method for antibodies from other classes or other organisms simply by designing reverse primers for the constant regions of their antibody of study.

## Materials and methods

### Hybridoma production and total RNA extraction

The hybridoma cells producing monoclonal antibodies were generated as described in [25]. Total RNA was extracted from the hybridoma cells using TRIzol Reagent (Invitrogen, 15596026) according to the manufacturer’s instructions.

### Reverse transcription to synthesize cDNA of antibody variable regions

The SMARTScribe Reverse Transcriptase kit from Clontech (Table 7) was used. Additionally used were the RNA samples (five hybridoma RNA samples from the mouse antibodies or one RNA sample from the chimeric antibody), primers (Tables 1 and 2 for mouse antibodies or Tables 5 and 6 for chimeric antibodies), 10 mM deoxynucleotide triphosphate mix (dNTPs), H_2_O, and an 80 U/μL RNAse inhibitor. Note: Make aliquots of the template-switch oligo and store at –80°C due to its RNA content.

**Table 7:** Kits and antibodies.

Purpose	Manufacturer: Catalog Number	Kit Contents or Antibody Details
Reverse Transcription	Clontech: 639537	5x SMARTScribe buffer 20 mM DTT 100 U/μL SMARTScribe Reverse Transcriptase
PCR Clean-Up and Gel Extraction	Macherey-Nagel: 740609.50	Buffers NTI, NT3, and NE DNA-binding columns
Blunt-End Cloning	Invitrogen: 450245	pCR-Blunt II-TOPO plasmid Salt solution (1.2 M NaCl, 0.06 M MgCl_2_) dNTP mix M13 forward and reverse sequencing primers
Miniprep	Macherey-Nagel: 740588.50	Buffers A1, A2, A3, AW, A4, and AE RNAse A DNA-binding columns
Effectene Transfection	Qiagen: 301425	Effectene Enhancer Buffer EC
Pierce Protein A Fab Preparation Kit	Thermo Fisher Scientific: 44985	Protein A beads IgG elution buffer (pH 2.8, amine-based)
Goat Anti-Human IgG Fc Antibody, Horseradish Peroxidase Conjugate, Affinity Purified	Thermo Fisher Scientific: A18817	Horseradish-peroxidase-conjugated polyclonal 2° antibody with specificity for human antibody Fc region Antibody Registry ID: AB_2535594
Peroxidase-Conjugated AffiniPure Goat Anti-Mouse IgG, Fcγ Fragment Specific	Jackson ImmunoResearch: 115-035-071	Horseradish-peroxidase-conjugated polyclonal 2° antibody with specificity for mouse antibody Fc region Antibody Registry ID: AB_2338506

Reverse transcription was executed according to the following protocol: All reactions were kept on ice during setup. For each RNA sample, three cDNA synthesis reactions were set up: one for the kappa chain, one for the lambda chain, and one for the heavy chain. Ideally, only one of the light chains will amplify per antibody. **1.** In PCR tubes, Mix #1 was prepared: 2 μL 50 ng/μL RNA, 1 μL 10 μM reverse RT primer based on antibody chain (e.g. mIGK RT, mIGL RT, or mIGHG RT for mouse antibodies), and 1 μL 10 mM dNTPs. For one RNA sample, three tubes of Mix #1 were needed, each containing a different reverse primer. **2.** In a 0.5 mL Eppendorf tube, Mix #2 was prepared: 1.95 μL H_2_O, 2 μL 5x SMARTScribe buffer, 1 μL 20 mM DTT, and 0.3 μL 100 μM template-switch oligo. Volumes given for Mix #2 are for one cDNA synthesis reaction, so scale-up occurred as necessary, i.e. three times the volumes for Mix #2 were prepared per hybridoma RNA sample. One master mix of Mix #2 was prepared for all reactions. **3.** Any RNA secondary structure was denatured by incubating the tubes containing Mix #1 at 72°C for 3 minutes in a thermocycler. **4.** During denaturation of Mix #1, the following was added to Mix #2: 0.25 μL 80 U/μL RNAse inhibitor and 0.5 μL 100 U/μL SMARTScribe Reverse Transcriptase per cDNA synthesis reaction. **5.** 6 μL of Mix #2 was added to each tube of denatured Mix #1. **6.** In the thermocycler, the combined mix was incubated at 42°C for 60 minutes, then at 70°C for 5 minutes to stop the reaction. The reactions were held at 4°C. PCR amplification was done immediately after reverse transcription. No cDNA purification step was necessary.

### PCR amplification of antibody variable regions

**1.** PCR reactions for each cDNA synthesis were set up: 10 μL 5x PCR buffer, 1 μL 10 mM dNTPs, 3 μL synthesized cDNA from the RT reaction, 2.5 μL 10 μM universal forward primer ISPCR, 2.5 μL 10 μM reverse PCR primer based on antibody chain (e.g. mIGK PCR, mIGL PCR, or mIGHG PCR for mouse antibodies), 30.5 μL H_2_O, and 0.5 μL 2 U/μL Phusion polymerase (or other high-fidelity polymerase). **2.** A touch-down/step-down PCR was performed according to the following thermocycler conditions: 98°C for 30 seconds; 10 cycles of 98°C for 15 seconds, 63–57.5°C for 30 seconds (decreasing the temperature by 0.5°C each cycle), and 72°C for 30 seconds; 15 cycles of 98°C for 15 seconds, 56°C for 30 seconds, and 72°C for 30 seconds; followed by 72°C for 7 minutes; and holding at 4°C. **3.** 5 μL of each RT-PCR reaction was run on a 1% agarose gel in TAE buffer at 90 V. The amplified mouse antibody products appeared between 550–600 base pairs. The amplified human antibody products appeared between 750–850 base pairs. The Quick Load Purple 2-Log DNA Ladder (NEB, N0550S) was used as the standard.

### Gel extraction and sequencing or PCR clean-up, blunt-end cloning, miniprep, and sequencing of antibody variable regions

The total volume of each RT-PCR reaction was run on a 1% agarose gel in TAE buffer at 90 V. Bands of interest were excised, and DNA was extracted from the gel using Macherey-Nagel’s PCR Clean-Up and Gel Extraction kit (Table 7). The extracted DNA was Sanger sequenced by Sequetech Corporation using the ISPCR primer (Table 2).

Alternatively, the RT-PCR reactions were PCR-cleaned using Macherey-Nagel’s PCR Clean-Up and Gel Extraction kit (Table 7). 2 μL of each PCR-cleaned product was blunt-end cloned into the pCR-Blunt-II-TOPO vector according to the blunt-end cloning kit manual (Table 7). Next, 3 μL of each TOPO cloning reaction was transformed into chemically competent *E*. *coli*. 100 μL of each transformation was spread on LB plates containing 50 μg/mL kanamycin and incubated at 37°C overnight. After obtaining colonies, 5–10 colonies per antibody chain were inoculated in 5 mL LB/kanamycin medium and grown at 37°C with 250 rpm shaking overnight. These cultures were miniprepped using Macherey-Nagel’s miniprep kit (Table 7) and the resulting plasmid DNA was Sanger sequenced by Sequetech Corporation using the M13 forward primer.

A custom-written Python program was used to identify amplicons and plasmid clones containing antibody variable region sequences. The sequences originating from the same *E*. *coli* transformation, corresponding to a specific chain from a specific antibody, were then aligned using Clustal Omega to check for sequence consensus of the antibody chain. The final DNA sequence for each antibody variable region was submitted to IgBLAST with default parameters and mouse selected as the organism for query sequence to determine percent identity to the IgBLAST reference sequences for light and for heavy chains. The DNA sequences were also submitted to IMGT/BlastSearch using default parameters to determine percent identity to the IMGT reference sequences.

### Expression and purification of the Spike 8 antigen

A synthetic gene codon-optimized for *E*. *coli* expression encoding the human astrovirus serotype 8 capsid spike protein amino acids 424 to 648 (Spike 8, UniProtKB entry Q9IFX1) was purchased from Integrated DNA Technologies. To make the Spike 8 expression plasmid, the gene was cloned into pET52b (Addgene) in-frame with a C-terminal thrombin cleavage site and a 10-histidine purification tag. The plasmid was verified by DNA sequencing. Next, the plasmid was transformed into *E*. *coli* strain BL21(DE3). Cultures were inoculated and grown in LB/ampicillin medium. At an optical density of 0.6, protein production was induced with 1 mM isopropyl-D-thiogalactopyranoside (IPTG) at 18°C for 18 hours. *E*. *coli* cells were lysed by ultrasonication in 20 mM Tris-HCl pH 8.0, 500 mM NaCl, and 20 mM imidazole (Buffer A) containing 2 mM MgCl_2_, 0.0125 U/μL benzonase (Merck Millipore, 71205), and 1x protease inhibitor cocktail set V EDTA-free (Merck Millipore, 539137). Proteins were batch purified from soluble lysates with TALON metal affinity resin (GE Healthcare, 28-9574-99) and eluted with Buffer A containing 500 mM imidazole. Proteins were dialyzed overnight into 10 mM Tris-HCl pH 8.0, 150 mM NaCl (TBS) and further purified in TBS by size exclusion chromatography on a Superdex 75 column.

### Expression and purification of chimeric mAb 2D9

A synthetic gene codon-optimized for insect cell expression containing the 2D9 kappa and heavy chain variable regions connected by a linker was ordered from Integrated DNA Technologies. The kappa and heavy chain variable regions were amplified separately and individually cloned by Gibson assembly into the two pCMV-VRC01 antibody backbone vectors for light and for heavy chains, which contain the constant regions of VRC01, a human anti-HIV antibody targeting the gp120 protein.[34–36] The resulting expression plasmids, pCMV-VRC01_2D9_kappa and pCMV-VRC01_2D9_heavy, contain the variable regions from the original mouse antibody 2D9 and the constant regions from a human IgG1 antibody under control of the human cytomegalovirus promoter. This same cloning procedure was accomplished with the four remaining antibodies. All plasmids were verified by DNA sequencing. Using the Effectene Transfection kit (Table 7), 2 μg of each of the 2D9 constructs was transiently co-transfected into HEK 293F cells (Thermo Scientific, R79007) obtained from a neighboring laboratory. The HEK 293F cells were seeded the day before at 0.5 x 10^6^ cells/mL in 10 mL FreeStyle 293 medium (Gibco, 12338018). After 8 days of incubation at 37°C with 5% CO_2_, chimeric mAb 2D9 was purified from the HEK 293F cell medium with Protein A beads (Table 7). Chimeric mAb 2D9 was eluted with IgG elution buffer (pH 2.8, amine-based), and the elution was immediately neutralized with 2.0 M Tris pH 8.0.

### Total RNA extraction from transfected HEK 293F cells

The extraction of RNA from HEK 293F cells transiently transfected with expression plasmids for chimeric mAb 2D9 was done according to the manufacturer’s protocol for using TRIzol Reagent (Invitrogen, 15596026) to extract total RNA from cells grown in a monolayer. 3 mL TRIzol Reagent was used per T75 flask seeded 8 days prior with 10 mL of 0.5 x 10^6^ HEK 293F cells/mL.

### Purification of mouse mAb 2D9

Mouse mAb 2D9 was purified from mouse ascites fluid with Protein G beads (Thermo Scientific, 20398). Mouse mAb 2D9 was eluted with IgG elution buffer (pH 2.8, amine-based), and the elution was immediately neutralized with 2.0 M Tris pH 8.0.

### SDS-PAGE gel comparing chimeric mAb 2D9 and mouse mAb 2D9

Each SDS-PAGE gel sample was prepared with 4 μg protein. For each monoclonal antibody, a reducing sample and a non-reducing sample was prepared. For the reducing samples, purified protein was mixed with 5x SDS-PAGE loading dye and boiled at 100°C for 7 minutes. For the non-reducing samples, purified protein was mixed with 5x loading dye not containing β-mercaptoethanol and not boiled. The protein samples were loaded on a pre-cast 4–12% Bis-Tris gel (Novex Life Technologies, NP0321BOX) and run in MES-SDS buffer at 140 V. The gel was stained by Coomassie Blue. The Precision Plus Protein Dual Color Standard (Bio-Rad, 1610374) was used as the standard.

### ELISAs comparing Spike 8 binding by chimeric mAb 2D9 and mouse mAb 2D9

Each point was performed in triplicate. 150 μL per well of Spike 8 at 5 μg/mL in phosphate-buffered saline (PBS) was incubated overnight at room temperature on two 96-well ELISA microtiter plates. As a control, 150 μL per well of 5 μg/mL bovine serum albumin (BSA) in PBS was also incubated overnight. The plates were then washed three times with PBS containing 0.05% Tween 20 (PBST). The wells were blocked by adding 150 μL of 5% BSA in PBS to each well and incubating at room temperature for 1 hour followed by three PBST washes. Chimeric mAb 2D9 and mouse mAb 2D9, the primary antibodies, were diluted to 5 μg/ml with 1% BSA in PBS. 150 μL of chimeric mAb 2D9 was added to wells in the first column of one ELISA plate and serially diluted 1:3 with 1% BSA in PBS. This serial dilution was repeated on the other ELISA plate with mouse mAb 2D9. As a control, three rows of 5 μg/mL Spike 8 on each plate were left without primary antibody; 150 μL of 1% BSA in PBS was added instead to the first wells of these rows and serially diluted 1:3. The plates were incubated for 1 hour at room temperature and then washed three times with PBST.

For the ELISA in which the primary antibody was chimeric mAb 2D9, the plate was incubated for 1 hour at room temperature with 150 μL per well of secondary antibody, a goat anti-human IgG Fc antibody conjugated to horseradish peroxidase (HRP) (Table 7) diluted 1:20,000 with 1% BSA in PBS. For the ELISA in which the primary antibody was mouse mAb 2D9, the plate was incubated for 1 hour at room temperature with 150 μL per well of secondary antibody, a goat anti-mouse IgG Fc antibody conjugated to HRP (Table 7) diluted 1:8,500 with 1% BSA in PBS. Then the plates were washed three times with PBST and developed by adding 150 μL of 0.4 mg/mL horseradish peroxidase substrate o-phenylenediamine dihydrochloride (OPD) (Thermo Scientific, 34006) in 0.05 M phosphate-citrate buffer (pH 5.0) with 0.015% hydrogen peroxide for 10 minutes at room temperature. The reactions were stopped by incubation with 150 μL of 2 N sulfuric acid for 10 minutes at room temperature. The absorbance was measured at 490 nm.

## Abbreviations

BSA: Bovine serum albumin
CDR: Complementarity-determining region
ELISA: Enzyme-linked immunosorbent assay
HEK 293F: Human embryonic kidney cell line
IgG1: Immunoglobulin G subclass 1
mAb: Monoclonal antibody
MMLV: Moloney murine leukemia virus
5’ RACE: Rapid amplification of 5’ cDNA end
RT-PCR: Reverse transcription polymerase chain reaction
SMART: Switching mechanism at 5’ end of RNA transcript
Spike 8: Recombinant capsid spike domain from human astrovirus serotype 8
